# Sensory feeding problems, pica-related behaviors, and sleep habits among children with autism spectrum disorder in palestine: a parent-reported cross-sectional study

**DOI:** 10.3389/fped.2026.1899150

**Published:** 2026-07-24

**Authors:** Suheir Sabbah, Sana M. Namoura, Hamzeh Yacoub, Zaid Yacoub, Rita Yacoub

**Affiliations:** 1 Psychology Department, Al-Quds University, Jerusalem, Palestine; 2 Faculty of Medicine, Al-Quds University, Jerusalem, Palestine; 3 Faculty of Medicine, Arab American University, Ramallah, Palestine; 4 SurgiCore-Jerusalem, Surgical Interest Group of Jerusalem, Faculty of Medicine, Al-Quds University, Jerusalem, Palestine

**Keywords:** autism spectrum disorder, cross-sectional study, feeding disorders, palestine, pica-related behaviors, sensory feeding problems, sensory processing, sleep habits

## Abstract

**Background:**

Autism Spectrum Disorder (ASD) is commonly associated with feeding difficulties and sleep disturbances, yet their co-occurrence remains understudied in Arab and Palestinian contexts. Pica and broader pica-related sensory feeding behaviors are among the most clinically significant feeding problems in children with ASD, carrying serious health risks. This study examined the level of parent-reported sensory feeding problems, pica-related behaviors, and sleep habit disturbances in children with ASD attending care centers in the West Bank, Palestine, and explored their association.

**Methods:**

A cross-sectional descriptive-correlational survey was conducted. A convenience sample of 105 parents of children with ASD was recruited from care centers in the West Bank during August 2025. Two instruments were used: a 20-item literature-informed Sensory Feeding/Pica-Related Behaviors Scale developed for this study, and a 21-item Sleep Habits Scale adapted from the Arabic scale developed and validated by Al-Merdasi. The feeding scale underwent expert content review and pilot testing, while internal consistency was assessed for both instruments in the present sample. The feeding scale was designed to describe caregiver-reported sensory feeding difficulties and pica-related behaviors rather than to establish a DSM-5-TR or ICD-11 diagnosis of pica. All sleep items were coded so that higher scores indicated greater sleep disturbance; sleep adequacy items were reverse-scored. Data were analyzed using descriptive statistics, independent-samples t-tests, one-way ANOVA with LSD *post-hoc* tests, and Pearson correlation in SPSS. Effect sizes were reported as Cohen's d for t-tests and partial *η*² for ANOVA.

**Results:**

Sensory feeding problems and Pica-related behaviors scores were at a moderate level overall (M = 1.946, SD = 0.307; relative weight 64.9%). Sleep habit disturbances were also moderate (M = 3.123, SD = 0.551; 62.5%), with sleep anxiety ranking highest (M = 3.507). No significant differences in Sensory feeding and pica-related behavior scores were found by any demographic variable. For sleep habits, significant differences were found by child age (F = 10.038, p = 0.001, *η*² = 0.164), with younger children appearing to score higher, though the oldest age group was small (n = 7) and the finding should be interpreted cautiously. Significant differences were also found by child sex (t = 2.195, p = 0.030, d = 0.43), with higher sleep disturbance scores among females. A significant positive moderate correlation was found between pica-related problem scores and sleep disturbance scores (r = 0.463, p < 0.001).

**Conclusions:**

This parent-reported cross-sectional study found moderate levels of sensory feeding problems, pica-related behaviors, and sleep disturbances among children with ASD in the West Bank, Palestine, and a significant positive association between them. Because the study used convenience sampling and researcher-developed instruments with preliminary validation only, findings should be interpreted as preliminary and should not be taken as estimates of clinically diagnosed pica or sleep disturbances. They highlight the importance of integrating screening for pica-like behaviors and sleep problems within ASD services, and support the need for future longitudinal studies incorporating clinical diagnosis, gastrointestinal assessment, nutritional biomarkers, and validated measures.

## Background

Autism Spectrum Disorder (ASD) is a complex neurodevelopmental condition defined by persistent deficits in social communication and interaction, alongside restricted, repetitive patterns of behavior, interests, or activities (1). Under the current DSM-5-TR framework, ASD is conceptualized as a single spectrum diagnosis differentiated by severity levels rather than discrete subtypes, reflecting the wide heterogeneity in clinical presentation across individuals (1). Global reviews now estimate that approximately 1 in 127 individuals are autistic, with substantial variation across studies, regions, and diagnostic practices (2). In high-income countries, prevalence estimates from national surveillance data have reached 1 in 36 children, reflecting improved detection and broadened diagnostic criteria (3). In the Arab region, including Palestine, systematic epidemiological data remain limited, though growing service utilization at autism care centers across the West Bank suggests a substantial and insufficiently documented burden of ASD in this population.

The neurobiology of ASD involves complex genetic, epigenetic, and environmental interactions that affect early brain development. Neuroimaging and genetic studies have consistently implicated disruptions in synaptic connectivity, cortical organization, and the development of social brain networks (4). These neurobiological disruptions have downstream effects on sensory processing, behavioral regulation, and homeostatic functions including appetite, gastrointestinal motility, and sleep architecture domains that are directly relevant to this study (5, 6).

Feeding difficulties are among the most frequently reported comorbidities in ASD, affecting an estimated 46%–89% of affected children across studies (7). These difficulties arise from multiple converging mechanisms. First, sensory processing differences are pervasive in ASD: children may exhibit hypersensitivity or hyposensitivity to gustatory, olfactory, tactile, and proprioceptive stimuli, resulting in strong food preferences or refusals, insistence on specific food textures, and an inability to tolerate novel foods (8). Second, the rigid and repetitive behavioral patterns characteristic of ASD often manifest as strong adherence to limited food repertoires and heightened distress when food routines are disrupted (1). Third, gastrointestinal (GI) dysfunction is common in ASD, estimated at 23%–70% across studies and contributes to discomfort-driven food avoidance and oral aversion (9). Together, these factors place children with ASD at significant risk for nutritional deficiencies, growth faltering, and atypical oral behaviors, including the ingestion of non-food substances.

Pica is defined by DSM-5-TR as the persistent ingestion of non-nutritive, non-food substances (e.g., paper, dirt, chalk, plastic, hair, ice) for a period of at least one month at a developmental level where such behavior is inappropriate, and is one of the most severe feeding-related problems observed in children with ASD (1). The International Classification of Diseases, 11th Revision (ICD-11) similarly requires that the behavior be persistent or severe enough to warrant clinical attention, cause harm or significant health risk, and be inconsistent with the individual's developmental level (10). Common subtypes include geophagia (soil and clay), pagophagia (ice, often associated with iron deficiency anemia), and amylophagia (raw starch), though the range of substances ingested in ASD is wide and often idiosyncratic (11). In the present study, however, the feeding instrument was not designed to diagnose DSM-5-TR or ICD-11 pica; rather, it was intended to capture a broader continuum of sensory feeding difficulties and pica-related behaviors reported by caregivers.

Population-based studies have reported pica behaviors in 23.5% of children with ASD compared with 3.5% among typically developing peers in the United States (5), and in 10–13.6% in the United Kingdom (6). A recent cross-sectional study from South India identified pica behaviors in 36.5% of children with ASD (12). The comorbidity of intellectual disability substantially elevates pica risk within ASD: studies consistently find higher pica rates among those with co-occurring intellectual disability relative to those with ASD alone (13, 14). Three primary etiological pathways have been proposed for pica in ASD (11, 15): (a) nutritional deficiency particularly in iron, zinc, and calcium which may create unconscious cravings for mineral-containing non-food substances; (b) sensory-seeking behavior driven by oral hyposensitivity and atypical sensory integration; and (c) behavioral reinforcement through attention, escape from demands, or automatic sensory reinforcement.

The health consequences of pica in ASD are serious and potentially life-threatening. Ingested materials may cause lead and heavy metal poisoning, intestinal obstruction and perforation, parasitic infection from geophagia, dental erosion, and respiratory tract obstruction (16, 17). Given these risks, early identification and intervention are essential. Behavioral interventions based on positive reinforcement, response blocking, differential reinforcement of alternative behaviors, and environmental modification are the most evidence-based approaches to pica management (18).

Sleep disturbances constitute a second major and pervasive comorbidity in ASD, affecting an estimated 50%–80% of children with the disorder, a rate two to three times higher than in typically developing children (19). The most commonly reported sleep problems in ASD include difficulty initiating sleep, frequent nocturnal awakenings, early morning waking, prolonged sleep latency, night terrors, and shortened overall sleep duration (20, 21). These disturbances are not merely secondary to behavioral or anxiety symptoms; they have independent neurobiological underpinnings including melatonin dysregulation, disrupted circadian rhythmicity, and GABAergic system dysfunction (22). Sensory hypersensitivity to light, noise, and tactile stimuli in the sleep environment further impedes sleep onset and maintenance (23).

The consequences of sleep problems in ASD extend well beyond nighttime hours. Inadequate sleep is associated with increased daytime irritability, heightened repetitive behaviors, reduced adaptive functioning, impaired learning and memory consolidation, and greater caregiver burden (24). Longitudinal evidence suggests that sleep problems in early childhood with ASD may predict worse behavioral outcomes in later childhood, underscoring the importance of early screening and intervention (25). The American Academy of Neurology (AAN) has issued a practice guideline specifically addressing the treatment of insomnia and disrupted sleep behavior in children and adolescents with ASD, recommending evidence-based behavioral sleep interventions as a first-line approach, with melatonin considered for cases of confirmed circadian rhythm dysfunction or sleep-onset difficulty unresponsive to behavioral strategies (19).

Although pica-related behaviors and sleep disturbances are individually well-documented in ASD, their co-occurrence and interrelationship have received limited research attention. Mechanistically, multiple pathways may link these two domains. First, pica behaviors frequently cause GI problems including abdominal pain, constipation, intestinal obstruction, and altered gut microbiome composition that are themselves strongly associated with sleep disruption in ASD (26). Second, nutritional deficiencies associated with pica, particularly iron-deficiency anemia and zinc deficiency, are known to impair the synthesis of melatonin and serotonin, both critical for sleep regulation (15). Iron deficiency is independently associated with restless legs syndrome and disrupted sleep architecture in pediatric populations (27). Third, the same sensory dysregulation that drives oral sensory-seeking behaviors (pica-like behavior) may also elevate general neurological arousal, impeding the transition to sleep. A study among Turkish children with ASD found that those with sleep disorders had significantly more gastrointestinal problems than those without (26), and a study among Egyptian children with ASD identified a significant positive association between pica behaviors and sleep disturbances (28).

In Palestine, where ASD services are unevenly distributed, early diagnosis remains delayed, and families frequently rely on caregiver-based management without adequate specialist support (29), understanding the overlap between pica-related behaviors and sleep problems carries direct service planning implications. No study has previously examined this relationship in a Palestinian context. Identifying the co-occurrence and association of these two comorbidities in this understudied population is an essential step toward developing targeted, contextually appropriate screening and intervention pathways within West Bank ASD care centers.

This study therefore aimed to: (1) determine the level of parent-reported sensory feeding problems and pica-related behaviors among children with ASD in the West Bank; (2) examine whether sensory feeding/pica-related behavior scores differ by child age, sex, parent-reported diagnostic label, caregiver identity, parental education, household income, or marital status; (3) describe sleep habit patterns across five domains; (4) examine whether sleep habit scores differ by the same variables; and (5) assess the correlation between sensory feeding/pica-related behavior scores and sleep disturbance scores.

## Methods

### Study design and setting

A cross-sectional descriptive-correlational survey design was employed. Reporting follows the Strengthening the Reporting of Observational Studies in Epidemiology (STROBE) guidelines (30). Data were collected in August 2025 from ASD care centers in the West Bank, Palestine.

### Participants and sampling

A convenience sample of 105 parents or primary caregivers of children with a formal ASD diagnosis was recruited from care centers in the West Bank. Inclusion criteria required: (a) primary caregiver of a child aged 2–18 years with a formal ASD diagnosis from a qualified professional; (b) written informed consent; and (c) ability to complete the questionnaire independently or with assistance. Caregivers returning incomplete responses were excluded.

Seven ASD care centers operating in the West Bank were approached; all seven agreed to participate. Questionnaires were distributed to approximately 120 eligible caregivers across all centers. Of these, 112 questionnaires were returned (response rate approximately 93%), of which 7 were excluded due to incomplete responses (≥30% missing items), yielding 105 complete, analyzable responses.

The sample size was determined pragmatically based on the number of eligible caregivers accessible through participating centers during the data collection period. *post-hoc* power analysis indicates that the sample was adequate to detect moderate correlations (r ≥ 0.27 at *α* = 0.05, power = 0.80, n = 105) but underpowered for certain subgroup comparisons, particularly the 10–18-year age group (n = 7). Ethical approval was obtained from Al-Quds University. Written informed consent was obtained from all participants, with anonymity and confidentiality assured. Table 1 presents sample characteristics.

**Table 1:** Demographic characteristics of the study sample (*N* = 105).

Variable	Category	*n*	%
Child Age	< 5 years	46	43.8
	5 to < 10 years	52	49.5
	10–18 years	7	6.7
Child Sex	Male	61	58.1
	Female	44	41.9
Parent-Reported Diagnostic Label^a^	Classic Autism	37	35.2
	Asperger Syndrome	28	26.7
	ASD (unspecified)	23	21.9
	Rett Syndrome	17	16.2
Primary Caregiver	Mother	88	83.8
	Father	17	16.2
Parental Education	University degree	57	54.3
	High school or below	34	32.4
	Postgraduate	14	13.3
Monthly Income	< 1,800 NIS	45	42.9
	1,800–< 3,500 NIS	35	33.3
	3,500–< 5,000 NIS	15	14.3
	≥ 5,000 NIS	10	9.5
Marital Status	Married	70	66.7
	Widowed	20	19.0
	Divorced	15	14.3

a Diagnostic information was recorded from centers or parent reports using legacy terminology. These categories do not map onto current DSM-5-TR ASD subtypes and were reported descriptively only.

### Instruments

#### Sensory feeding and pica-related behaviors scale

A 20-item researcher-developed scale assessed care-giver reported sensory feeding difficulties and pica-related behaviors in children with ASD. The scale was informed by DSM-5-TR (1) and relevant literature (6, 13). However, the instrument was not intended to establish a clinical diagnosis of pica**;** rather, it was designed to capture a broader continuum of feeding-related difficulties that included non-food ingestion behaviors as well as sensory and behavioral features potentially associated with pica.

The scale comprised two subscales: Eating-Related Pica Behaviors (15 items: non-food ingestion, sensory food refusal, smell-discrimination difficulty, atypical oral behaviors); and Behavioral Pica-Related Features (5 items: urge intensity, concealment, functional impact). Items were rated on a three-point scale (1 = No, 2 = Unsure, 3 = Yes). Total scores are interpreted as low (<1.67), moderate (1.67–2.33), or high (>2.33). Internal consistency in a pilot sample of 15 parents was satisfactory (*α* = 0.765 overall; subscales: 0.764 and 0.705).

#### Scale development process

Both study instruments were developed in Arabic by the research team after reviewing DSM-5-TR/ICD-11 diagnostic descriptions, published studies on feeding problems and pica-related behaviors in ASD, and literature describing common sleep problems in children with ASD. Items were drafted to reflect caregiver-observable behaviors relevant to Palestinian children attending ASD care centers. Draft items were reviewed by a panel of 10 specialists in psychology, special education, and educational measurement for clarity, cultural appropriateness, and content relevance. Items identified by two or more reviewers as unclear, redundant, or conceptually inappropriate were revised or removed. The revised questionnaire was pilot-tested with 15 caregivers to assess comprehensibility and feasibility before use in the main study.

#### Sleep habits scale

A 21-item researcher-developed scale adapted from Almerdasi assessed five sleep habit domains: sleep anxiety (5 items), morning awakening difficulty (4 items), sleep adequacy (4 items), sleep-onset latency/breathing problems (3 items), and night terrors (5 items). A researcher-developed scale was used because no locally validated Arabic sleep instrument specific to Palestinian children with ASD was available; items were based on common sleep domains reported in ASD literature and reviewed by specialists (20, 21), including bedtime anxiety or sleep-onset dependency, difficulty waking in the morning, insufficient or poor-quality sleep, delayed sleep onset, possible breathing-related sleep symptoms, and night-time arousal/terror-like episodes.

The scale was designed to capture caregiver-reported sleep difficulties rather than to establish formal ICSD-3 sleep diagnoses such as insomnia disorder, obstructive sleep apnea, circadian rhythm sleep–wake disorder, or parasomnias. Items were rated on a five-point frequency scale (1 = Never to 5 = Always). All items were coded so that higher scores indicate greater sleep disturbance; sleep adequacy items were reverse-scored accordingly. Domain means are interpreted as low (<2.34), moderate (2.34–3.67), or high (>3.67). Internal consistency was acceptable (*α* = 0.781 overall; domain alphas: 0.704–0.856).

#### Validity

Content validity of the feeding scale was established through expert review by a panel of 10 specialists in special education, psychology, and educational measurement from Palestinian universities. Items agreed for revision or deletion by two or more reviewers were amended. Internal consistency validity was supported by Pearson item–domain correlations (all p < 0.01). The sleep scale was adapted from a previously validated Arabic scale, and its internal consistency was reassessed in the present sample. Accordingly, both instruments were used to generate descriptive caregiver-reported indices, not diagnostic classifications.

Data were analyzed using SPSS version 25.0. Descriptive statistics (means, SDs, relative percentages) were computed for all scales and subscales. Group differences were examined using independent-samples t-tests for two-group variables and one-way ANOVA for variables with three or more groups; subgroup analyses were considered exploratory because of multiple comparisons and small cell sizes. Effect sizes are reported as Cohen's d for t-tests and partial *η*² for ANOVA (small ≈ 0.01, medium ≈ 0.06, large ≈ 0.14). Subgroup analyses were exploratory and were not powered to establish equivalence between demographic groups; therefore, non-significant findings were interpreted as absence of detected differences rather than evidence of no true differences. The Pearson product-moment correlation assessed the sensory feeding/ pica-related behaviors scores and sleep disturbance scores. Statistical significance was set at *α* = 0.05 (two-tailed).

## Results

### Sensory feeding problems and pica-related behaviors

Parents rated their children's sensory feeding problems and pica-related behaviors at a moderate level overall (M = 1.946, SD = 0.307; relative weight 64.9%). The Eating-Related Feeding/Pica Behaviors subscale scored slightly higher (M = 1.955, SD = 0.291; 65.2%) than the Behavioral Pica-Related Features subscale (M = 1.920, SD = 0.507; 64.0%), both at moderate levels. Table 2 presents selected item-level results for each subscale.

**Table 2:** Descriptive statistics for pica-related problem subscales and selected items (*N* = 105).

Scale/Item	M	SD	Wt (%)	Level
Eating-Related Feeding/Pica Behaviors (subscale)	1.955	0.291	65.2	Moderate
Highest: Inability to discriminate food by smell	2.162	0.878	72.1	Moderate
2nd: Eating very small quantities	2.114	0.874	70.5	Moderate
Lowest: Ingests non-food items ≥ 1 month (DSM-5-TR criterion)	1.762	0.838	58.7	Moderate
Behavioral Pica-Related Features (subscale)	1.920	0.507	64.0	Moderate
Highest: Strong urge to consume non-food materials	2.162	0.942	72.1	Moderate
Lowest: Attempts to conceal behavior	1.524	0.761	50.8	Moderate
Overall Sensory Feeding and Pica-Related Behaviors Scale	1.946	0.307	64.9	Moderate

Selected items shown; full item-level data available from the corresponding author on request.

The highest-rated eating-related item was inability to discriminate food by smell (M = 2.162), followed by eating very small quantities (M = 2.114) and indiscriminate oral approach to food stimuli (M = 2.076). The item most directly reflecting DSM-5-TR-like pica feature -persistent ingestion of non-food substances for at least one month- received the lowest mean (M = 1.762). This pattern suggests that the scale captured broader sensory feeding difficulties and oral/pica-related behaviors more strongly than clinically defined pica-like ingestion behavior *per se***.** In the Behavioral subscale, the highest-rated item was strong urge to consume non-food materials (M = 2.162); the lowest was attempts to conceal the behavior (M = 1.524).

### Group differences in sensory feeding/pica-related behavior scores

Table 3 presents group comparisons for sensory feeding/pica-related behavior scores. No statistically significant differences were found for any demographic variable, suggesting that pica-related and sensory feeding problems were similarly distributed across the demographic variables studied.

**Table 3:** Group differences in sensory feeding/Pica-related behavior scores by demographic variable (*N* = 105).

Variable	Groups [M (SD)]	Stat	df	p	ES	Sig.
Child Age	<5: 1.960 (0.229) 5–10: 1.934 (0.363) 10–18: 1.950 (0.335)	F = 0.087	2,102	0.916	*η*²=0.002	ns
Child Sex	Male: 1.906 (0.334) Female: 2.001 (0.256)	t = 1.569	103	0.120	d = 0.32	ns
Primary Caregiver	Mother: 1.928 (0.317) Father: 2.038 (0.228)	t = 1.356	103	0.178	d = 0.38	ns
Parental Education	Univ: 1.929 (0.317) Postgrad: 1.846 (0.316) HS: 2.016 (0.278)	F = 1.738	2,102	0.181	η²=0.033	ns
Household Income	4 groups; M range 1.850–1.993	F = 0.917	3,101	0.435	η²=0.026	ns
Marital Status	Married: 1.918 (0.346) Divorced: 1.940 (0.252) Widowed: 2.050 (0.141)	F = 1.459	2,102	0.237	η²=0.028	ns

### Sleep habit disturbances

The mean sleep disturbance score was 3.123 (SD = 0.551) on a 1–5 scale, suggesting a moderate descriptive range of caregiver-reported sleep difficulties. Table 4 presents domain-level results. Sleep anxiety was the highest-rated domain (M = 3.507, SD = 0.720; 70.1%), followed by morning awakening difficulty (M = 3.431, SD = 0.843; 68.6%), sleep-onset latency/breathing problems (M = 3.016, SD = 1.006; 60.3%), night terrors (M = 3.004, SD = 0.825; 60.1%), and sleep adequacy (M = 2.567, SD = 0.631; 51.3%). All items were coded so that higher scores indicate greater sleep disturbance; sleep adequacy items were reverse-scored.

**Table 4:** Descriptive statistics for sleep habit domains (*N* = 105).

Domain	M	SD	Rel. Weight (%)	Level
Sleep Anxiety	3.507	0.720	70.1	Moderate
Morning Awakening Difficulty	3.431	0.843	68.6	Moderate
Sleep-Onset Latency/Breathing	3.016	1.006	60.3	Moderate
Night Terrors	3.004	0.825	60.1	Moderate
Sleep Adequacy (reverse-scored)	2.567	0.631	51.3	Moderate
Overall Sleep Habits Scale	3.123	0.551	62.5	Moderate

Within sleep anxiety, the most prevalent items were moving to another person's bed during the night (M = 3.810; high level) and needing a parent present to fall asleep (M = 3.686; high level). Within morning awakening difficulty, taking a long time to wake up in the morning was rated highest (M = 3.848; high level).

### Group differences in sleep habit scores

Table 5 presents group comparisons for sleep habit scores.

**Table 5:** Group differences in sleep habit scores by demographic variable (*N* = 105).

Variable	Groups [M (SD)]	Stat	df	p	ES	Sig.
Child Age^a^	<5: 3.290 (0.391) 5–10: 3.075 (0.576) 10–18: 2.388 (0.651)	F = 10.038	2,102	0.001	η²=0.164	*
Child Sex	Male: 3.025 (0.575) Female: 3.260 (0.488)	t = 2.195	103	0.030	d = 0.43	*
Primary Caregiver	Mother: 3.127 (0.571) Father: 3.106 (0.446)	t = 0.138	103	0.891	d = 0.04	ns
Parental Education	Univ: 3.143 (0.566) Postgrad: 3.252 (0.580) HS: 3.038 (0.514)	F = 0.824	2,102	0.442	η²=0.016	ns
Household Income	4 groups; M range 3.091–3.219	F = 0.204	3,101	0.893	η²=0.006	ns
Marital Status	Married: 3.074 (0.588) Divorced: 3.229 (0.480) Widowed: 3.217 (0.455)	F = 0.838	2,102	0.435	η²=0.016	ns

a Overall ANOVA indicated age-group variation; interpret cautiously because the 10–18-year group was small (*n* = 7) and subgroup analyses were exploratory.*Statistically significant at *p* < 0.05; *ns* = not significant.

An exploratory ANOVA showed age-group variation in sleep disturbance scores (F = 10.038, *p* = 0.001, *η*² = 0.164). This finding should be interpreted cautiously because the oldest group comprised only seven participants and multiple subgroup analyses were conducted. The age effect was significant for sleep anxiety, morning awakening difficulty, and sleep adequacy domains, but not for sleep-onset/breathing or night terror domains.

Overall sleep scores differed significantly by child sex (t = 2.195, p = 0.030, d = 0.43, moderate effect), with higher sleep disturbance scores among females (M = 3.260, SD = 0.488) relative to males (M = 3.025, SD = 0.575). The sex difference was significant for the sleep-onset/breathing domain (t = 2.912, p = 0.004) but not the other four domains. No significant differences were found by diagnostic label, caregiver identity, parental education, household income, or marital status.

### Correlation between feeding/pica-related behaviors and sleep habits

Table 6 presents the exploratory unadjusted Pearson correlations between sensory feeding/pica-related behavior scores and the sleep habit domains. Exploratory unadjusted correlation analyses showed positive associations between sensory feeding/pica-related behavior scores and all sleep habit domains. The overall sensory feeding/pica-related behavior score correlated moderately with overall sleep disturbance (r = 0.463, *p* < 0.001). Domain-level correlations for the overall sensory feeding/pica-related behavior score ranged from r = 0.324 for difficulty falling asleep within 20 min to r = 0.422 for sleep adequacy problems. The eating-related feeding/pica subscale showed its strongest correlation with sleep adequacy problems (r = 0.436), whereas the behavioral pica-related features subscale showed its strongest correlation with night terrors (r = 0.465). These findings should be interpreted as exploratory and unadjusted.

**Table 6:** Exploratory unadjusted correlations between sensory feeding/pica-related behavior scores and sleep habit domains (*N* = 105).

Feeding/pica-related domain	Sleep anxiety	Morning awakening difficulty	Sleep adequacy problems/reverse-scored	Difficulty falling asleep	Night terrors	Overall sleep disturbance
Eating-related feeding/pica behaviors	0.295**	0.360**	0.436**	0.243*	0.307**	0.387**
Behavioral pica-related features	0.305**	0.399**	0.430**	0.366**	0.465**	0.452**
Overall sensory feeding/pica-related behaviors	0.336**	0.421**	0.422**	0.324**	0.410**	0.463**

Correlations are exploratory, unadjusted Pearson correlations.

*p < 0.05.

**p < 0.01.

## Discussion

### Principal findings

This study provides the first Palestinian evidence on the co-occurrence and interrelationship of caregiver-reported sensory feeding problems, pica-related behaviors, and sleep disturbances in children with ASD. Both constructs were observed at moderate levels, suggesting that these are relevant concerns in this sample, although the cross-sectional parent-reported design does not allow conclusions about clinical management or severity trajectories. The central finding was a significant positive correlation (r = 0.463) between sensory feeding/pica-related behavior scores and sleep disturbance scores.

### Sensory feeding problems and pica-related behaviors

The moderate level of parent-reported pica-related behaviors is consistent with prior findings in comparable populations. Abd Rabbo and Abd Al-Aziz (31) similarly reported moderate pica severity in Egyptian children with ASD. The predominance of items such as inability to discriminate food by smell, eating very small quantities, and indiscriminate oral approach to food stimuli suggests that the feeding scale primarily captured sensory feeding difficulties and broader oral/pica-related behaviors, rather than clinically defined pica alone (8). Notably, the item most closely aligned with DSM-5-TR pica criteria -persistent ingestion of non-food substances- showed the lowest mean score in the eating-related subscale. Accordingly, the present findings should not be interpreted as estimating the prevalence of DSM-5-TR or ICD-11 pica, but rather as describing a broader pattern of feeding-related difficulties and pica-associated behaviors in this sample.

The item most directly reflecting DSM-5-TR pica criteria received the lowest mean (M = 1.762), suggesting the scale captured a broader feeding difficulty construct. The sensory feeding problems and pica-related behaviors Scale should therefore be validated against clinician-rated assessments in future research, and findings should not be interpreted as the prevalence of clinically diagnosed pica. This pattern aligns with evidence that pica behaviors in ASD exist on a continuum with broader oral sensory-seeking behaviors rather than as a discrete, binary phenomenon (13, 14).

The absence of significant pica-related score differences across all demographic variables is consistent with prior literature (5, 13, 31), suggesting that pica-related and sensory feeding behaviors may not be strongly patterned by the measured sociodemographic variables in this sample. This uniformity may also partly reflect the relatively homogeneous convenience sample and limited between-group variability.

### Sleep habit disturbances

Sleep anxiety was the most prevalent domain, with children frequently moving to others' beds and requiring parental presence to sleep. This pattern has been documented in children with ASD and may reflect difficulties in emotional self-regulation and sleep-onset independence (20). Morning awakening difficulty ranked second, consistent with evidence of delayed sleep phase and prolonged sleep inertia in ASD (21).

Sleep disturbance scores appeared higher among younger children; however, because the oldest group comprised only seven participants, this finding must be interpreted cautiously and requires replication with adequate subgroup sizes. The large effect size (*η*² = 0.164) is noteworthy and consistent with evidence that sleep problems in ASD are highly prevalent and behaviorally challenging in early childhood (19, 24). Females showed significantly higher sleep disturbance scores than males (d = 0.43), particularly in the sleep-onset/breathing domain. This may reflect higher internalizing anxiety in females with ASD (32) and the additional burden imposed by social camouflaging, which is more common in females with ASD (33–35).

### Association between sensory feeding/pica-related behaviors and sleep

The significant positive correlation (r = 0.463) replicates the direction of findings by Mohamed et al. (28) in Egyptian children with ASD, and is consistent with mechanistic pathways described in the literature. Pica behaviors frequently result in GI problems that are independently associated with sleep disruption in ASD (26). Iron and zinc deficiencies associated with pica impair melatonin synthesis and sleep regulation (15, 27). Heightened sensory-seeking activity driving pica-like behaviors may also reflect a broader arousal pattern that impedes sleep initiation. However, because the present study did not measure GI symptoms, nutritional biomarkers, ASD severity, medication use, or sensory processing directly, these pathways remain speculative. The association may be consistent with shared behavioral or physiological pathways including sensory dysregulation, gastrointestinal discomfort, or nutritional deficiencies which should be directly assessed in future research.

### Clinical implications

Although the findings are preliminary, they suggest several possible implications for ASD care in the West Bank. First, medical evaluation including hematological testing for iron and zinc deficiency should be considered when clinically indicated, particularly in children with persistent pica-like behaviors, restricted diets, symptoms of anemia, or GI complaints. Second, behavioral intervention programs applying positive reinforcement, response blocking, differential reinforcement of alternative behaviors, and environmental modification should be integrated into ASD care (17, 18). Third, given the high prevalence of sleep anxiety, caregiver education programs should include evidence-based behavioral sleep interventions consistent bedtime routines, sleep environment adaptations for sensory sensitivities, and graduated parental withdrawal techniques as the first-line approach, consistent with the AAN practice guideline for ASD sleep (19). Pharmacologic options such as melatonin should be considered, under clinician guidance, for confirmed circadian rhythm dysfunction or sleep-onset difficulty unresponsive to behavioral strategies (19). The integration of pica-like behavior and sleep screening into routine ASD assessments would facilitate earlier identification and referral.

### Limitations

Several limitations should be noted. First, convenience sampling from seven centers limits generalizability; replication using stratified sampling across the West Bank is needed. Second, exclusive reliance on parent report is subject to recall and social desirability bias. Third, the cross-sectional design precludes causal inference. Fourth, the 10–18-year age group comprised only seven participants. Fifth, diagnostic labels reflect older DSM-IV terminology and cannot be treated as current DSM-5-TR subtypes. Sixth, the pica scale includes both pica-specific and broader sensory feeding items, and findings should not be equated with the prevalence of clinically diagnosed pica. Seventh, instruments underwent only preliminary validation. Eighth, and most importantly, the study did not assess key covariates including ASD severity, intellectual disability, language impairment, medication use, GI symptoms, nutritional biomarkers (ferritin, iron, zinc), epilepsy, screen time, co-sleeping practices, and behavioral therapy exposure. Future studies should adjust for these factors to clarify whether the pica–sleep association is independent or mediated. Ninth, subgroup analyses were exploratory, involved multiple comparisons, and included small cell sizes; therefore, these findings should not be interpreted as confirmatory.

## Conclusions

This parent-reported cross-sectional study found moderate levels of sensory feeding problems and pica-related behaviors and sleep disturbances among children with ASD attending care centers in the West Bank, Palestine, and a significant positive moderate association between them (r = 0.463). Sleep anxiety was the most prevalent sleep concern. Pica-related behavior and sleep disturbance profiles did not differ significantly by most sociodemographic variables, suggesting these problems may not be strongly patterned by the measured demographic factors in this sample. Younger children and females showed higher sleep disturbance scores, though the small oldest age group limits confidence in the age comparison. Because the study used convenience sampling, researcher-developed instruments with preliminary validation only, and cross-sectional parent-reported data, findings should be interpreted as preliminary. They highlight the importance of integrating screening for pica-related behaviors, sensory feeding problems, and sleep disturbances within ASD services in the West Bank, and support the need for future longitudinal studies incorporating clinical diagnosis, gastrointestinal assessment, nutritional biomarkers, validated sleep measures, and rigorous control for key covariates.

## Data Availability

The raw data supporting the conclusions of this article will be made available by the authors, without undue reservation.
